# The TonB‐Dependent Transport System Facilitates the Uptake of Inorganic Metal Mediators in *Pseudomonas putida* KT2440 in a Bioelectrochemical System

**DOI:** 10.1111/1751-7915.70206

**Published:** 2025-08-03

**Authors:** Anna Weimer, Jens Krömer, Bin Lai, Christoph Wittmann

**Affiliations:** ^1^ Institute of Systems Biotechnology Saarland University Saarbrücken Germany; ^2^ Systems Biotechnology Group Helmholtz Centre for Environmental Research—UFZ Leipzig Germany; ^3^ BMBF Junior Research Group Biophotovoltaics Helmholtz Centre for Environmental Research—UFZ Leipzig Germany

**Keywords:** 2‐ketogluconate, bioelectrochemical system, extracellular electron transfer, ferricyanide, mediator transport, *Pseudomonas putida* KT2440, redox mediator, TonB

## Abstract

Mediator‐based extracellular electron transfer (EET) in a bioelectrochemical system is a unique approach to regulate the microbial redox and energy metabolism of *Pseudomonas putida* KT2440, which enables a new‐to‐nature high product yield under anaerobic conditions. Previous studies identified respiratory complex III in the inner membrane as a key redox protein involved in mediator (ferricyanide) interactions, but the exact mechanism through which the mediator crosses the outer membrane to extract electrons from membrane‐bound redox proteins and transfer them to the anode remains unclear. In this study, we demonstrated the critical role of the TonB‐dependent system, a widespread transportation system in gram‐negative bacteria, in the mediator‐based EET process. Transcriptomic analyses revealed significant upregulation of TonB‐dependent receptors in response to ferricyanide exposure, suggesting their involvement in mediator uptake. Deletion of the TonB complex resulted in *a* > 50% decrease in the mediator reduction rate and current output, confirming the role of the TonB‐dependent system in mediator transport. Additionally, increasing passive diffusion through the overexpression of the general porin OprF increased cell permeability and the mediator reduction rate, but it failed to compensate for the absence of TonB‐dependent transport. These findings suggest that both systems act in a complementary manner: the TonB‐dependent system is likely the primary mechanism for periplasmic mediator uptake, whereas OprF is likely involved mainly in mediator efflux. Further bioelectrochemical system experiments demonstrated that, with a functional TonB‐dependent system, OprF overexpression increased current output, glucose consumption, and 2‐ketogluconate production, suggesting a viable strategy for enhancing the efficacy of mediator‐based EET. This work reveals the major mediator transport mechanism in *P. putida* and deepens the understanding of the mediator‐based EET pathway, laying the basis for future rational engineering of EET kinetics and facilitating the integration of mediator‐based electron transfer into industrial biotechnology to push its process boundaries.

## Graphical abstract

Mediator‐based extracellular electron transfer in *Pseudomonas putida* KT2440 relies on the TonB‐dependent system for effective mediator (ferricyanide) transport. Transcriptomic and functional analyses confirmed its key role, while OprF overexpression enhanced membrane permeability and current output. These findings clarify mediator uptake mechanisms and support rational engineering to improve anaerobic bioelectrochemical production processes.

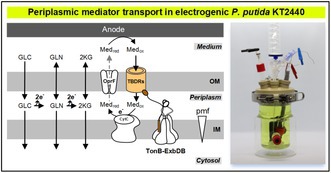

## 1 Introduction

Converting the strictly aerobic *Pseudomonas putida* KT2440 (Weimer et al. 2020) into an organism capable of anaerobic metabolism has long been a goal for expanding its industrial applications (Nikel and de Lorenzo 2013; Steen et al. 2013; Kampers et al. 2019, 2021). Notably, *P. putida* can maintain anaerobic metabolism growth within a bioelectrochemical system (BES) supplemented with hydrophilic redox mediators such as [Fe(CN)_6_]^3−/4^ and [Co(bipy)_3_]. In this system, the anode functions as an alternative terminal electron acceptor to oxygen, enabling anaerobic activity for several weeks (Lai et al. 2016; Yu et al. 2018; Nguyen et al. 2021; Pause et al. 2023; Weimer et al. 2024). This adaptation relies on mediator‐based extracellular electron transfer (EET), a widely used method in microbial electrochemical systems. It is used to increase electron exchange between microbes and electrodes, which can increase bioenergy generation (Ucar et al. 2017), environmental remediation (Li et al. 2017), and electricity‐driven bioproduction (Fruehauf et al. 2020; Virdis et al. 2022). More importantly, it induces the electrogenic activity of many nonmodel electrogens (Vassilev et al. 2018; Gu et al. 2023; Sun et al. 2023) and has the potential to integrate microbial electrochemical technology into industrial biotechnology.

In *P. putida* , mediator‐based EET provides an external driving force for redox reactions, enabling high‐yield biochemical synthesis that is not achievable through conventional microbiological approaches (Lai et al. 2016; Yu et al. 2018; Nguyen et al. 2021; Pause et al. 2023; Weimer et al. 2024). Efficient electron shuttling *between cells and the electrode* via *a redox mediator* results in a closed‐loop process cycle, which requires three critical steps: (i) transport of the mediator into the periplasm to access the redox protein, (ii) reduction of the mediator by the redox protein, and (iii) transport of the mediator out of the periplasm to the electrode for reoxidation. The molecular mechanism of mediator‐based EET in *P. putida* was partially revealed in our previous work (Lai et al. 2020): inhibition of aerobic respiration complex III, that is, cytochrome c reductase, completely blocked the current output, suggesting its important role in the EET process by bridging the intracellular electron transfer network to the redox mediator. As cytochrome c reductase is an integral membrane protein located on the inner membrane, the mediator must access this protein spatially, likely representing the rate‐limiting step in the EET process (Gemünde et al. 2022).

There are a limited number of studies on periplasmic mediator transport, with most studies focusing on natural mediators. The heterologous expression of the general porin OprF from *Pseudomonas aeruginosa* has been shown to increase the electrogenic activity of *Escherichia coli* cultivated in a riboflavin‐mediated microbial fuel cell, suggesting the involvement of passive diffusion. However, the selective transport of phenazine derivatives, widely studied self‐secreted mediators from *P. aeruginosa* (Rabaey et al. 2005), has also been reported (Chukwubuikem et al. 2021), indicating the importance of active transport systems for natural mediators. In contrast, research on the transport of artificial mediators, mostly inorganic metal complexes, has been lacking. Artificial mediators offer a promising and controllable approach to establish EET pathways between nonmodel electrogens, including both heterotrophs and photoautotrophs, and electrodes.

In this work, we aimed to identify the major transport mechanisms of inorganic metal mediators in *P. putida* KT2440. Expression profiling under BES conditions with ferricyanide as the mediator revealed a key role of TonB‐dependent active transport in periplasmic mediator uptake, which we investigated in addition to porin‐mediated passive diffusion. This work enhances our understanding of the mediator‐based EET pathway and provides a solid foundation for the rational engineering and optimisation of EET kinetics, as well as for transferring this system into a wide range of electrogenic microbes, including industrially relevant microbial strains.

## 2 Results

### 2.1 Analysis of Outer Membrane Gene Expression Revealed That TonB‐Dependent Receptors Play a Key Role in Periplasmic Mediator Transport

**FIGURE 1:**
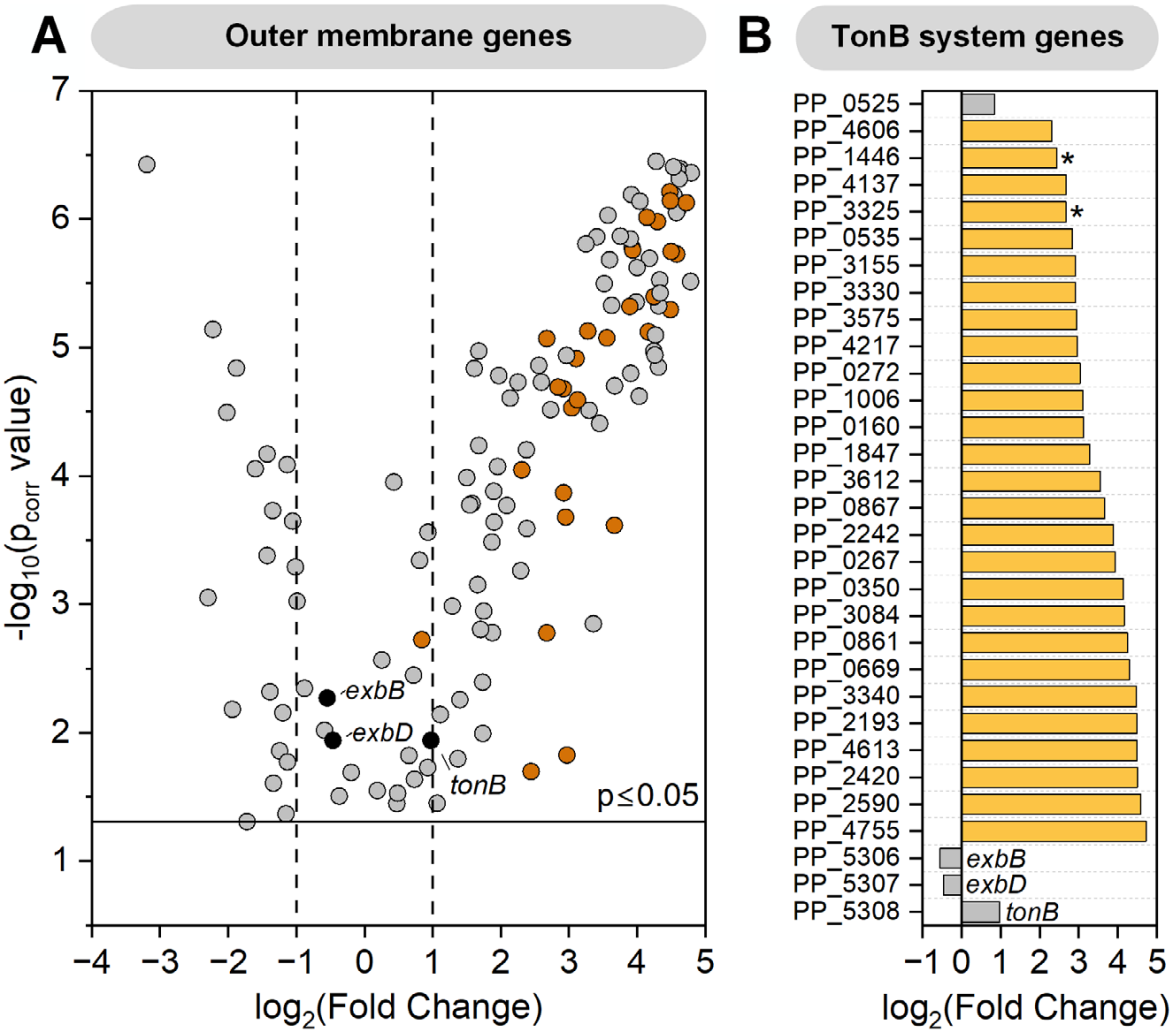
Differential gene expression of outer membrane‐localised genes (*p*_corr_ ≤ 0.05). (A) Volcano plot depicting changes in the expression of outer membrane genes. TonB‐dependent receptors (TBDRs) are highlighted in orange, whereas *tonB*, *exbB*, and *exbD* are marked in black. (B) Log_2_‐fold changes in TBDRs and *tonB*, *exbB*, and *exbD* (*p*_corr_ ≤ 0.05). Significantly upregulated genes (log_2_‐FC ≥ 2, *p*_corr_ ≤ 0.05) are shown in orange. TBDRs with significantly greater abundances in the proteomic dataset are marked with (PP_1446: Log_2_‐FC: 1.63, PP_3325: Log_2_‐FC: 1.53). The cells were collected after 24 h of bioelectrochemical cultivation and compared to samples taken at the start of the process (0 h) (*n* = 4).

**FIGURE 2:**
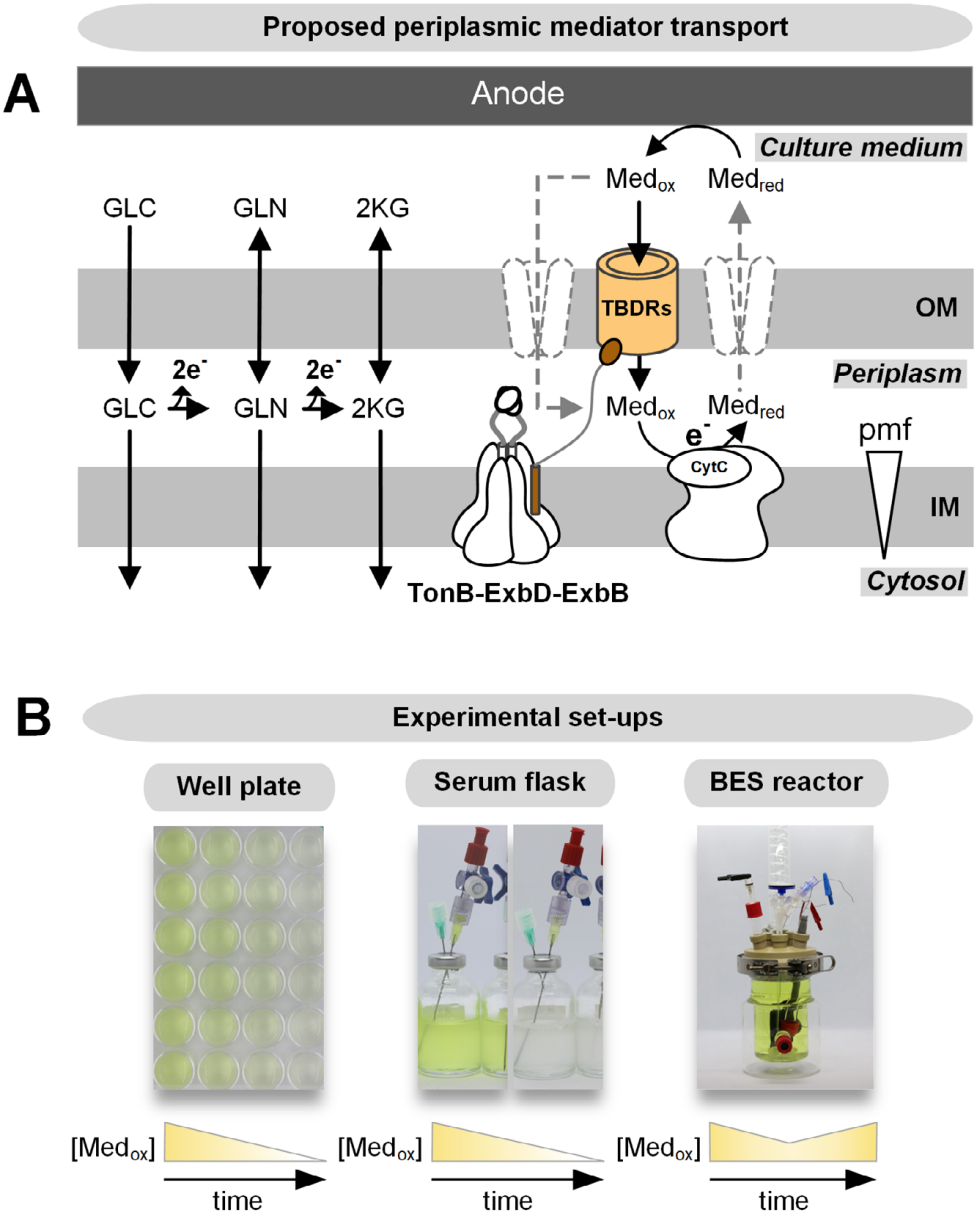
Proposed model for mediator transport across the outer membrane and applied screening methods. (A) Schematic representation of TonB‐dependent transport. The TonB complex (TonB‐ExbD‐ExbB), which is localised at the inner membrane, uses the proton motive force (pmf) to transduce energy to TBDRs (orange) at the outer membrane via TonB (brown), enabling substrate‐specific uptake. TBDRs are proposed to facilitate the periplasmic uptake of oxidised mediators (Med_ox_, [Fe(CN)_6_]^3−^). In the periplasm, the mediator is reduced (Med_red_, [Fe(CN)_6_]^4−^) at the cytochrome c reductase as the key extracellular electron transfer site (Lai et al. 2020). (B) Experimental approaches to investigate mediator reduction rates and electron transfer, including cell culture plate assays, serum flask cultivation and bioelectrochemical system experiments. Unlike bioelectrochemical systems, where mediators are continuously reoxidized at the anode, the mediator undergoes complete reduction over time in serum flasks and cell culture plates.

A total of 149 genes were identified, with approximately half showing significant upregulation after 24 h of bioelectrochemical cultivation using 1 mM ferricyanide as the mediator, compared with that at the start of the process (0 h) (log2‐fold change (FC) ≥ 2, *p* ≤ 0.05) (Table S1). Notably, the preculture had no prior exposure to the mediator ferricyanide, indicating that the observed gene expression changes were induced specifically under BES conditions. Among the 74 significantly upregulated genes, 15 encode porins or porin‐like proteins, 6 are linked to RND (Resistance‐Nodulation‐Division) efflux pumps, and 9 encode proteins of unknown function. Notably, 27 of these genes belong to the TonB‐dependent receptor (TBDR) family (Figure 1A,B), despite the cells being in a nongrowth, energy‐starved state under anaerobic conditions (Weimer et al. 2024). The genome of *P. putida* KT2440 contains 30 TBDRs. These receptors, which are widespread in gram‐negative bacteria (Nikaido 2003), are active transporters that mediate substrate‐specific uptake into the periplasm. This transport is driven by the proton motive force transmitted by the TonB complex (*tonB, exbB, exbD*) localised at the inner membrane (Figure 2A) (Ratliff et al. 2022). While primarily associated with iron uptake (Klebba et al. 2021), TBDRs also contribute to drug and solvent tolerance (Godoy et al. 2001) and transport diverse substrates, such as saccharides (Bolam and van den Berg 2018) and lignin‐derived aromatics (Fujita et al. 2019), underscoring their functional versatility. Although *tonB, exbB*, and *exbD* were not significantly upregulated (Figure 1B), the significant upregulation of nearly all TBDRs suggested that the TonB system plays a key role in periplasmic mediator uptake. In support of this, two TBDRs (*PP_1446* and *PP_3325*) were significantly more abundant at the proteome level (Log_2_‐FC: 1.63 and 1.53, respectively) (Figure 1B).

The role of passive diffusion (porin‐dependent) in mediator transport has been demonstrated in *E. coli* with riboflavin as the mediator (Yong et al. 2013) and has been suggested in *Cupriavidus necator* with ferricyanide as the mediator (Gemünde et al. 2024). However, the involvement of the TonB system in periplasmic mediator transport remains unreported. A key determinant of transmembrane transport is how cells respond to the mediator. Interestingly, exposure to ferricyanide not only induced TBDR expression but also upregulated genes associated with metal homeostasis, including *czcC‐II* (*PP_2408*, Log_2_‐FC 4.8), *czcC* (*PP_5385*, Log_2_‐FC 4.6), *czcCI* (*PP_0045*, Log_2_‐FC 3.9), *copBI* (*PP_2204*, Log_2_‐FC 4.0), and *ompQ* (*PP_4211*, Log_2_‐FC 4.2) (Table 1). These findings suggest that *P. putida* can recognise ferricyanide and possibly other inorganic mediators as metal‐related stimuli, which activate metal metabolism. Although ferricyanide is generally not used by bacteria as an iron nutrient because its iron is tightly bound within the stable cyanide complex, rendering it biologically inaccessible, the cells may still perceive these mediators in two ways: either as valuable metal‐containing compounds (nutrients) that need to be taken up and/or as potentially harmful stressors that require detoxification. This dual perception is common in bacteria, where metal ions or metal complexes play essential biochemical roles but can become toxic at high concentrations.

**TABLE 1:** Strains and plasmids.

Strain and plasmid	Description	References
*E. coli*		
DH5α λpir	Host for plasmid amplification: *supE44*, *ΔlacU169* (*φ80 lacZΔM15*), *hsdR17* (*rk* ^−^ *mk* ^+^), *recA1*, *endA1*, *thi1*, *gyrA*, *relA*, *λpir* lysogen	Biomedal Life Sci., Seville, Spain
CC118λpir	Mating donor strain: *Δ(ara‐leu)*, *araD*, *ΔlacX174*, *galE*, *galK*, *phoA*, *thi1*, *rpsE*, *rpoB*, *argE (Am)*, *recA1*, lysogenic *λpir*	Biomedal Life Sci., Seville, Spain
HB101	Mating helper strain: *SmR*, *hsdR‐M* ^+^, *pro*, l*eu*, *thi*, *recA*	(Sambrook et al. 1989)
*P. putida*		
KT2440	Wild type *P. putida* KT2440	(Nelson et al. 2002)
pSEVA6213S	KT2440 pSEVA6213S (control strain)	This work
*ΔexbBD ΔtonB*	*ΔPP_5306–5308* pSEVA6213S	This work
*ΔPP_1446*	*ΔPP_1446*	This work
*ΔPP_3325*	*ΔPP_3325*	This work
*ΔPP_1446 ΔPP_3325*	*ΔPP_1446 ΔPP_3325*	This work
KT2440/234	*P. putida* KT2440 pSEVA234	This work
KT2440/234‐oprF	*P. putida* KT2440 pSEVA234‐oprF	This work
*ΔexbBD ΔtonB*/243	*P. putida ΔexbBD ΔtonB* pSEVA6213S pSEVA234	
*ΔexbBD ΔtonB*/243‐oprF	*P. putida ΔexbBD ΔtonB* pSEVA6213S pSEVA234‐oprF	This work
Plasmid		
pGNW2	Suicide plasmid for integration/deletion: *Km* ^*R*^, *oriR6K, lacZα* with two flanking I‐SceI sites, P 14 g → msfGFP	(Wirth et al. 2020)
pSEVA6213S	Helper plasmid: *Gm* ^*R*^, *oriV* (RK2), *xylS*, PEM7 → I‐SceI	(Wirth et al. 2020)
pSEVA234	*Km* ^*R*^; oriV (RK2), lacIq‐Ptrc	
pSEVA234‐oprF	pSEVA234 bearing oprF (PP_2089)	This study

### 2.2 Iron Supplementation Restored Growth in the *ΔexbBD ΔtonB* Mutant

**FIGURE 3:**
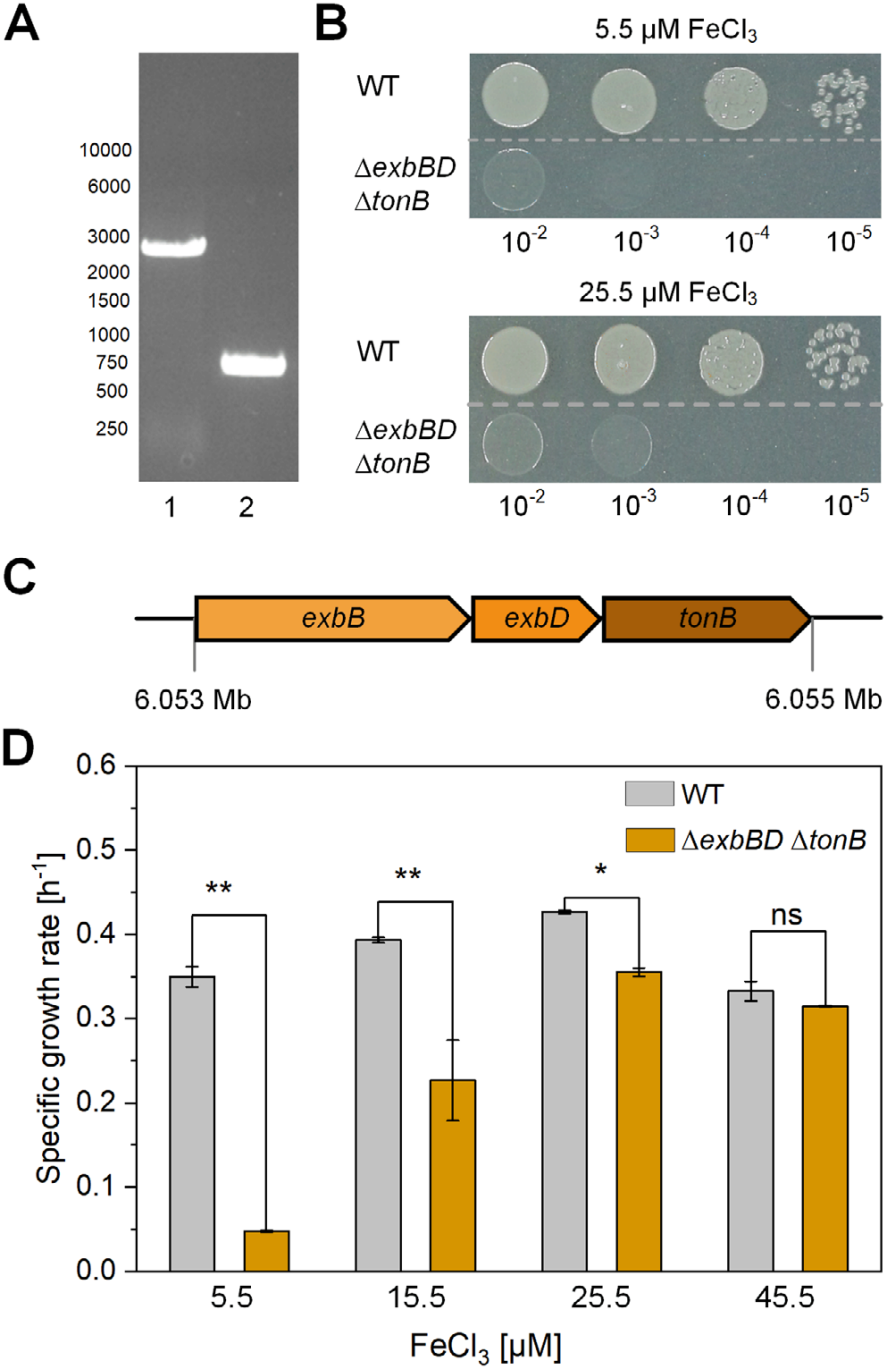
Growth differences between *P. putida* and its *ΔexbBD ΔtonB* mutant on LB plates supplemented with various iron concentrations. (A) Agarose gel analysis showing the successful deletion of *exbBD* and *tonB*, indicated by a 2123‐bp size difference between Lane 1 (wild type) and Lane 2 (Δ*exbBD* Δ*tonB* mutant). (B) Dilution plating of the wild‐type (WT) and *ΔexbBD ΔtonB* (*Δ*) strains on LB plates supplemented with different iron concentrations. (C) Specific growth rates (h^−1^) of wild‐type *P. putida* (grey bars) and its *ΔexbBD ΔtonB* mutant (orange bars) in DM9 medium supplemented with FeCl_3_ (10, 20, 40 μM) compared with the basal FeCl_3_ concentration of 5.5 μM. The values represent the means of biological triplicates (*n* = 3). Statistical significance was assessed using Student's *t* test (*p* > 0.05, not significant (ns); *p* < 0.05 (*); *p* < 0.01 (**)).

To investigate the role of the TonB complex in mediator transport, we generated a mutant strain of *P. putida* with an in‐frame deletion of the *exbB, exbD*, and *tonB* genes (*PP_53065308*) (Figure 3A), which encodes the core complex that is responsible for TBDR activation. Notably, unlike other Pseudomonads, such as *P. aeruginosa* , which harbours multiple TonB systems (Zhao and Poole 2000), *P. putida* KT2440 possesses only a single TonB complex that energises all TBDRs. The encoding genes are closely clustered in the genome (Figure 3C), suggesting that they form an operon and are cotranscribed, as proposed in other *P. putida* strains (Bitter et al. 1993). Consistent with previous studies, the *ΔexbBD ΔtonB* mutant presented a significant defect in aerobic growth (Figure 3B) (Poole et al. 1996; Godoy et al. 2001, 2004), highlighting the essential role of the TonB complex in nutrient acquisition, particularly iron uptake (Kümmerli 2023).

To assess whether iron supplementation could mitigate this growth defect, we cultured the mutant in minimal DM9, which contains a baseline FeCl_3_ concentration of 5.5 μM. Under these conditions, the growth rate of the mutant strain was approximately sevenfold lower than that of the wild‐type strain (Figure 3D). However, the addition of 20 μM FeCl_3_ restored growth, increasing the growth rate of the mutant to approximately 80% of that of the wild‐type strain. Interestingly, further increasing the FeCl_3_ concentration to 40 μM inhibited the growth of both the mutant and wild‐type strains (Figure 3D), suggesting potential iron toxicity at higher concentrations. These findings highlight the important role of the TonB complex in iron acquisition and emphasise the necessity of iron supplementation when the *ΔexbBD ΔtonB* mutant is cultured under nutrient‐limited conditions.

### 2.3 TonB Complex Deletion Impaired Periplasmic Transport and Extracellular Electron Transfer

The omics analyses suggested that the TonB‐dependent transport system plays a role in mediator uptake; thus, its deletion is expected to affect mediator reduction (i.e., the EET rate or current output in the BES). Restricted periplasmic mediator transport limits the availability of the mediator at the molecular interaction site, which is essential for facilitating EET from the cell to the mediator and, ultimately, to the anode. To investigate the role of TBDRs in mediator transport, we examined mediator turnover in *P. putida* with and without a functional TonB complex (*ΔexbBD ΔtonB*). Two experimental approaches were used (Figure 2B). The first approach involved anaerobic cultivation assays, where cells were grown in well plates and serum flasks with ferricyanide as a mediator. Over time, ferricyanide ([Fe(CN)_6_]^3−^), which is yellow, was reduced to ferrocyanide ([Fe(CN)_6_]^4−^), which is colourless. This reduction process was recorded via spectrophotometry. The second approach utilised a bioelectrochemical system. Unlike static cultivation, the bioelectrochemical system allows for continuous reoxidation of the mediator at the anode, extending the cultivation period. This enables complete oxidation of the substrate and provides a more detailed analysis of the resulting product profile. Additionally, the current signal generated by mediator reoxidation can be monitored in real time, offering a direct measure of the electron transfer efficiency.

**FIGURE 4:**
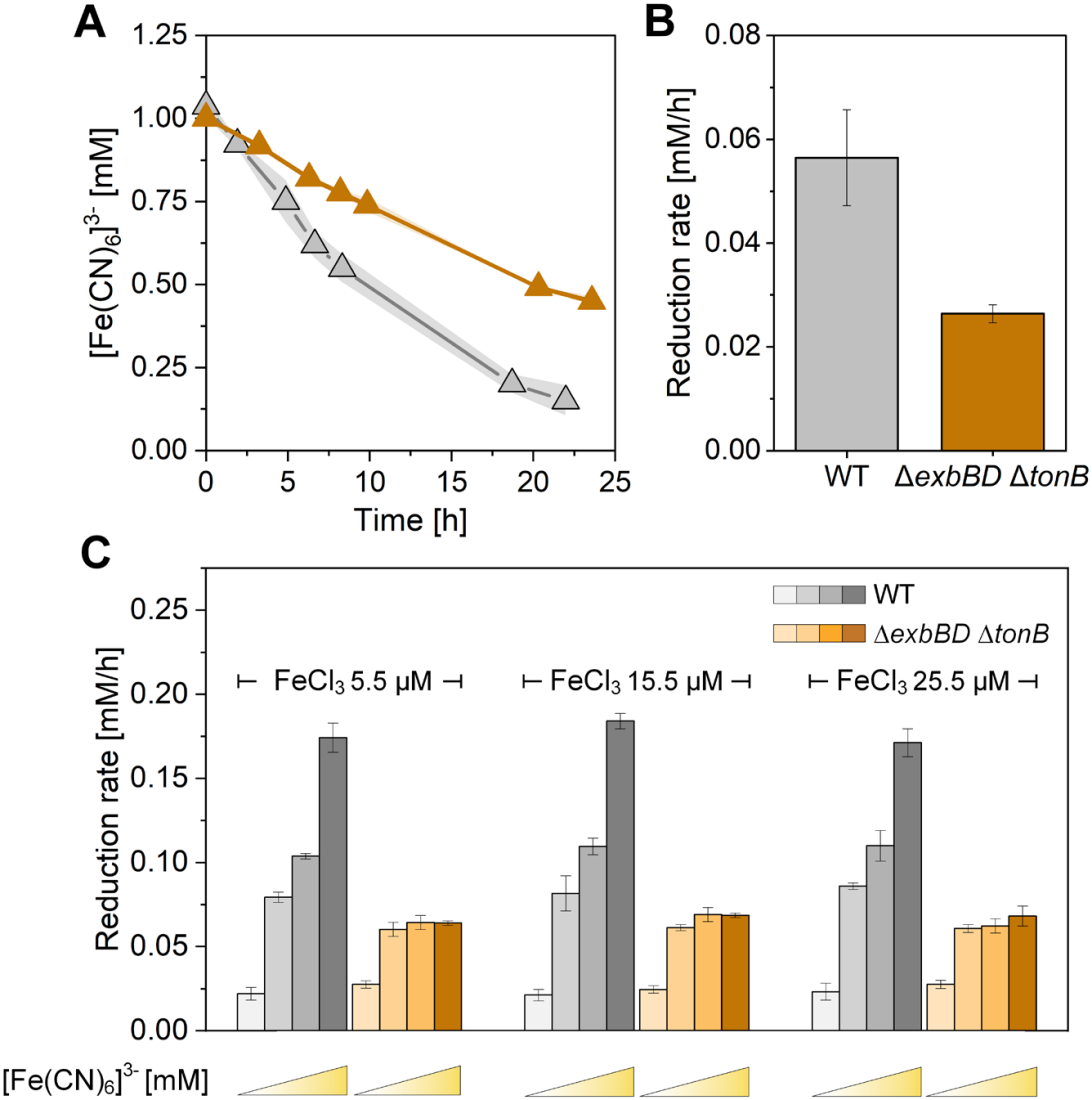
Differences in [Fe(CN)_6_]^3−^ reduction rates between *P. putida* and its *ΔexbBD ΔtonB* mutant. (A) Reduction of [Fe(CN)_6_]^3−^ (1 mM) in anaerobic serum flasks in wild‐type *P. putida* (grey) and its *ΔexbBD ΔtonB* mutant (orange). (B) Corresponding reduction rates of [Fe(CN)_6_]^3−^ in the serum flasks. (C) Reduction rates in well plates across different [Fe(CN)_6_]^3−^ concentrations (0.5, 1.0, 2.0 and 4.0 mM) and FeCl_3_ concentrations (5.5, 15.5 and 25.5 μM) (*n* = 3).

First, the reduction rates of the oxidised mediator ([Fe(CN)_6_]^3−^) were evaluated in wild‐type *P. putida* and its *ΔexbBD ΔtonB* mutant under anaerobic conditions using serum flasks and well plates (Figure 4). Compared with the wild‐type strain (0.056 ± 0.009 mM/h), the *ΔexbBD ΔtonB* strain presented a significantly lower mediator reduction rate (0.026 ± 0.001 mM/h) (Figure 4A,B). This finding supports the hypothesis that TonB‐dependent transport is involved in cross‐membrane mediator uptake. Unlike the observed growth defect under aerobic conditions (Figure 3), supplementing the medium with additional FeCl_3_ (10 μM, 20 μM) had no effect on the mediator reduction rate in either strain, which may also be due to a reduced cellular iron requirement in the absence of growth under anaerobic conditions (Figure 4C).

Given that *P. putida* has 30 TonB‐dependent receptors (TBDRs), identifying the specific TBDR(s) responsible for mediator uptake is important. As an initial step, we constructed single and double deletion mutants for *PP_1446* and *PP_3325*, two TBDRs that were significantly upregulated in the transcriptome and proteome datasets (Weimer et al. 2024). However, their deletion did not affect the mediator reduction rate compared with that of the wild type (Figure S1). These findings suggest that multiple TBDRs may contribute to mediator uptake, potentially exhibiting functional redundancy or overlapping roles. A more refined and targeted approach will be necessary to identify the specific TBDRs involved in mediator transport, paving the way for a deeper understanding of TonB‐dependent extracellular electron transfer in *P. putida* .

The reduced yet detectable mediator reduction rate in *P. putida ΔexbBD ΔtonB* (Figure 4A,B) indicates that an alternative transport mechanism may contribute to mediator uptake alongside the TonB‐dependent system. To investigate the potential role of passive diffusion through porins, we examined how varying mediator concentrations affected reduction rates.

In wild‐type *P. putida* , the mediator reduction rate increased steadily with increasing mediator concentration (0.5, 1.0, 2.0 and 4.0 mM). However, in the *ΔexbBD ΔtonB* mutant, the mediator reduction rate differed. While the reduction rate doubled when the mediator concentration increased from 0.5 to 1.0 mM, further concentration increases did not lead to a proportional increase in the reduction rate. Instead, the rate plateaued (Figure 4C). Notably, the residual activity observed in the TonB‐deficient strain was not due to the production of endogenous redox mediators. Previous studies have shown that in the absence of added mediators or heterologous expression of genes encoding phenazine biosynthesis, natural mediators produced by *P. aeruginosa* and *P. putida* exhibit negligible electrochemical activity (Schmitz et al. 2015; Lai et al. 2016). The observed plateau was unexpected, as passive diffusion via porins typically follows a concentration gradient: higher mediator concentrations should lead to increased periplasmic concentrations, thereby increasing EET rates in both strains. This result suggests that passive diffusion alone is insufficient to fully compensate for the loss of TonB‐dependent transport and plays only a minor role in mediator uptake. This could indicate that additional regulatory factors, limited porin‐mediated transport efficiency, or alternative transport pathways influence mediator transport.

### 2.4 Reduced Anaerobic Performance of *P. putida ΔexbBD ΔtonB* With Different Inorganic Metal Mediators in a BES

**FIGURE 5:**
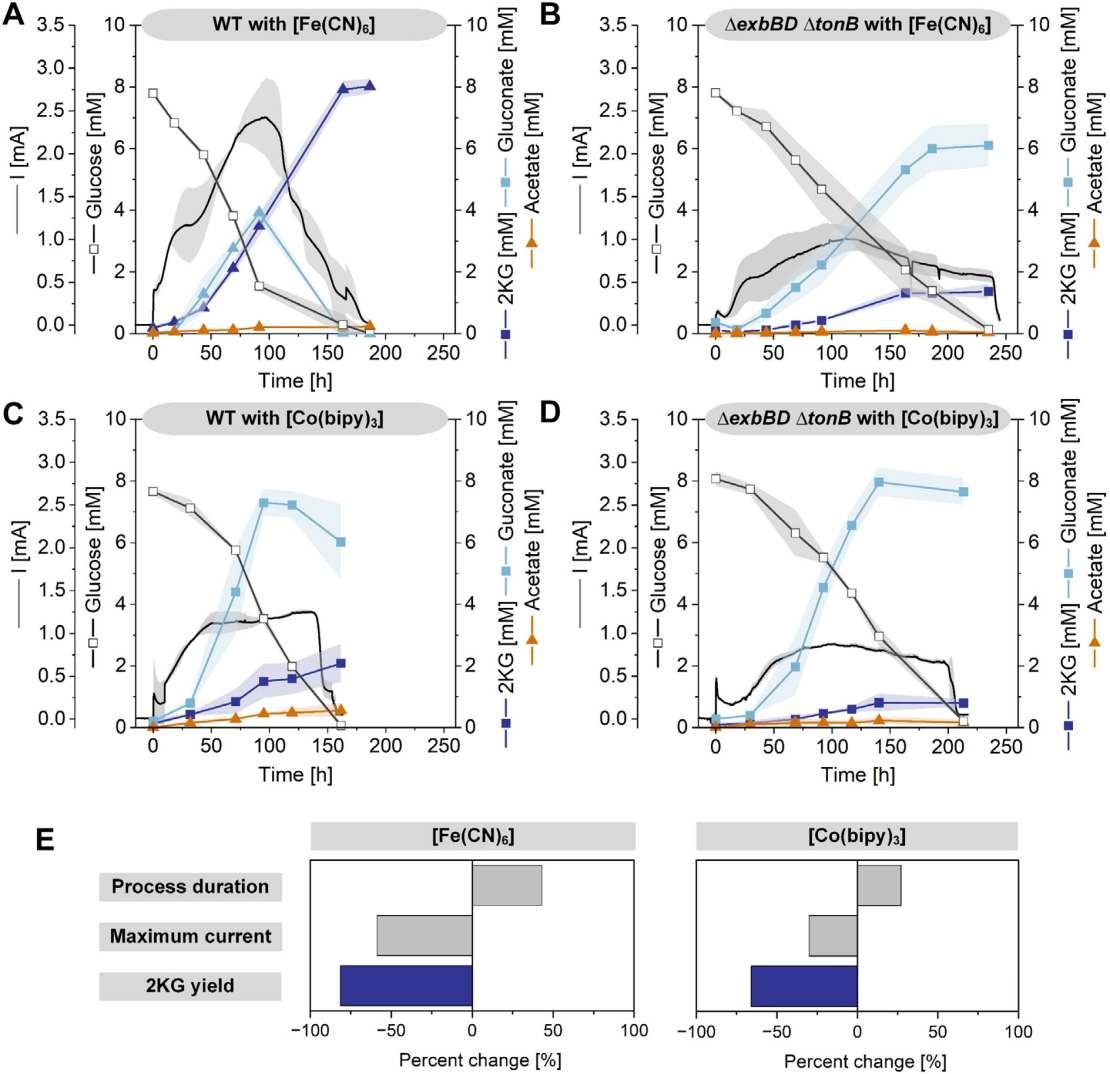
Bioelectrochemical performance of *P. putida* and its *ΔexbBD ΔtonB* mutant grown on glucose. Current (mA) and concentrations of glucose (mM), gluconate (mM), and 2‐ketogluconate (mM) during the cultivation of *P. putida* KT2440 and its *ΔexbBD ΔtonB* mutant using [Fe(CN)_6_]^3−^ (A, B) or [Co(bipy)_3_]^3+^ (C, D) as the mediator in a BES. (E) Summary of overall process performance to compare key metrics including the process duration, maximum current output, and 2‐ketogluconate yield, expressed as a percentage change in *P. putida ΔexbBD ΔtonB* relative to wild‐type *P. putida* KT2440. The data represent biological triplicates (*n* = 3).

The anaerobic performance of wild‐type *P. putida* and its *ΔexbBD ΔtonB* mutant was compared in a bioelectrochemical system to (1) evaluate the impact of TonB‐dependent transport on the utilisation of different inorganic metal mediators, specifically [Fe(CN)_6_]^3−/4‐^ and [Co(bipy)_3_]^3+/2+^, which have distinct redox potentials, and (2) analyse the resulting product spectrum when glucose is used as the substrate. When wild‐type *P. putida* was cultivated in a bioelectrochemical system with glucose and ferricyanide as the mediators, gluconate and 2ketogluconate (2KG) were initially produced as the main metabolic products. However, by the end of fermentation (186 h), gluconate was fully converted into 2KG, leaving 2KG as the main product (Figure 5A). Interestingly, a similar product profile was observed in the *ΔexbBD ΔtonB* mutant, but with a notable shift in product distribution. The mutant accumulated higher levels of the more reduced gluconate (degree of reduction: 3.67), whereas the conversion rate to more oxidised 2KG (degree of reduction: 3.33) was reduced. Furthermore, compared with the wild type, the mutant presented a greater than 50% reduction in current peak values and a prolonged fermentation duration, extending for approximately 60 h (Figure 5A,B,E). Since glucose oxidation to gluconate generates two electrons, whereas oxidation to 2KG yields four electrons, the observed shift towards gluconate in the *ΔexbBD ΔtonB* mutant suggests restricted periplasmic access to the mediator as an electron acceptor. This limitation likely arises from impaired mediator transport across the outer membrane. Notably, increasing the mediator concentration partially restored the current output (Figure S2).

When wild‐type *P. putida* was cultivated with [Co(bipy)_3_]^3+/2+^ as the mediator, lower levels of 2KG were produced, with gluconate emerging as the main product by the end of fermentation. This was accompanied by a lower current peak value than that of cultures cultivated with ferricyanide (Figure 5A,C). These findings show that [Fe(CN)_6_]^3−/4‐^, which has a higher formal redox potential than [Co(bipy)_3_]^3+/2+^ (Lai et al. 2016), is more effective in facilitating electron transfer. In the *ΔexbBD ΔtonB* mutant, the use of [Co(bipy)_3_]^3+/2+^ as the mediator led to a shift in the product spectrum, with an increased accumulation of gluconate, a reduced peak current, and an extended fermentation duration of approximately 50 h (Figure 5C–E). This pattern closely mirrored the results obtained with ferricyanide, further supporting the role of the TonB complex in the periplasmic transport of diverse inorganic metal mediators.

### 2.5 The TonB Complex Synergistically Enhances Extracellular Electron Transfer When Porin OprF Is Overexpressed

Increasing mediator concentrations in the medium led to higher mediator reduction rates (Figure 4B), suggesting that in addition to TonB‐dependent transport, passive diffusion through porins may also facilitate mediator transport across the outer membrane. In support of this hypothesis, the molecular masses of [Fe(CN)_6_]^3−/4^ (211.97 Da) and [Co(bipy)_3_]^3+/2+^ (527.53 Da) were below the exclusion threshold of OprF (< 3 kDa) (Hancock et al. 1979; Nestorovich et al. 2006), the primary nonspecific porin in pseudomonads (Sugawara et al. 2006). To further investigate the role of passive diffusion, OprF (*PP_2089*) was overexpressed using the IPTG‐inducible vector pSEVA234. Porin overexpression has previously been shown to increase current output in *E. coli* within a microbial fuel cell (Yong et al. 2013). Using a similar approach, we investigated whether strengthening passive diffusion through OprF overexpression could increase the current output in *P. putida* and its *ΔexbBD ΔtonB* mutant. This allowed us to assess whether increased porin‐mediated transport could partially compensate for the absence of TonB‐dependent transport in facilitating mediator uptake and electron transfer.

**FIGURE 6:**
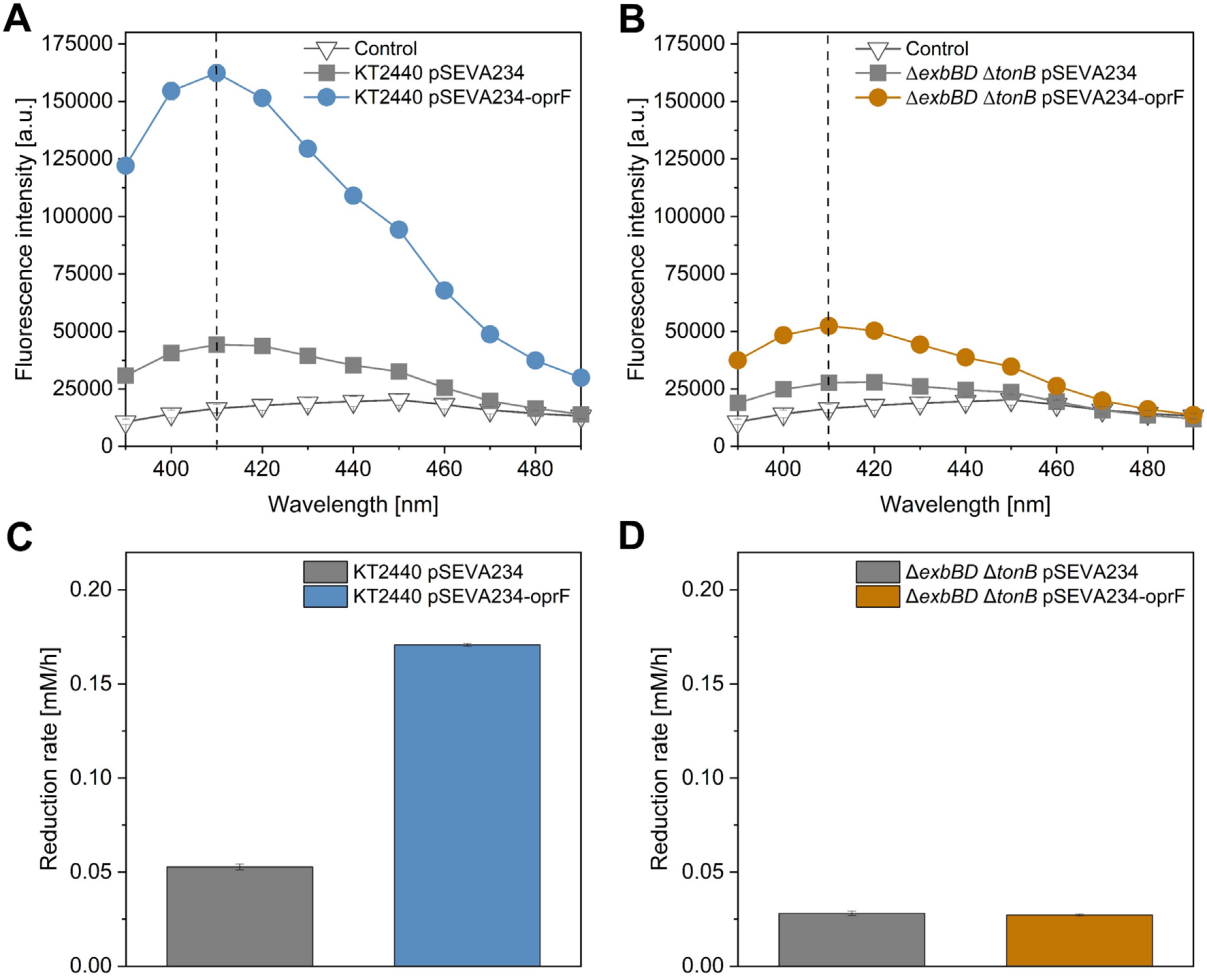
Effects of OprF overexpression on cell membrane permeability and the [Fe(CN)_6_]^3−^ reduction rate. (A) NPN uptake assay measuring membrane permeability in *P. putida* pSEVA234 (grey squares), *P. putida* pSEVA234‐oprF (blue circles), and a control without cells (white triangles). (B) NPN uptake in *P. putida ΔexbBD ΔtonB* pSEVA234 (grey squares), *P. putida ΔexbBD ΔtonB* pSEVA234‐oprF (orange circles), and a control without cells (white triangles). (C) Anaerobic serum flask assay measuring [Fe(CN)_6_]^3−^ reduction rates in *P. putida* pSEVA234 (grey) and *P. putida* pSEVA234‐oprF (blue). (D) Corresponding reduction rates in *P. putida ΔexbBD ΔtonB* pSEVA234 (grey) and *P. putida ΔexbBD ΔtonB* pSEVA234‐oprF (orange).

To investigate the impact of OprF overexpression on cell permeability, a 1Nphenylnaphthylamine (NPN) uptake assay was performed (Figure 6A,B). NPN is a fluorescent probe that has a weak fluorescence intensity in aqueous environments but becomes highly fluorescent in hydrophobic environments (Träuble and Overath 1973). The outer leaflet of the bacterial outer membrane is highly structured and is primarily composed of hydrophilic lipopolysaccharide (LPS), which acts as a barrier against hydrophobic molecules, including NPN (Nikaido 1989). However, when the integrity of the outer membrane is compromised, either through porin overexpression or membrane damage, NPN can penetrate the membrane and interact with its hydrophobic regions, leading to a significant increase in fluorescence intensity. This makes the NPN uptake assay a valuable tool for assessing changes in membrane permeability and integrity (Hancock and Wong 1984; Loh et al. 1984; Helander and Mattila‐Sandholm 2000).

**FIGURE 7:**
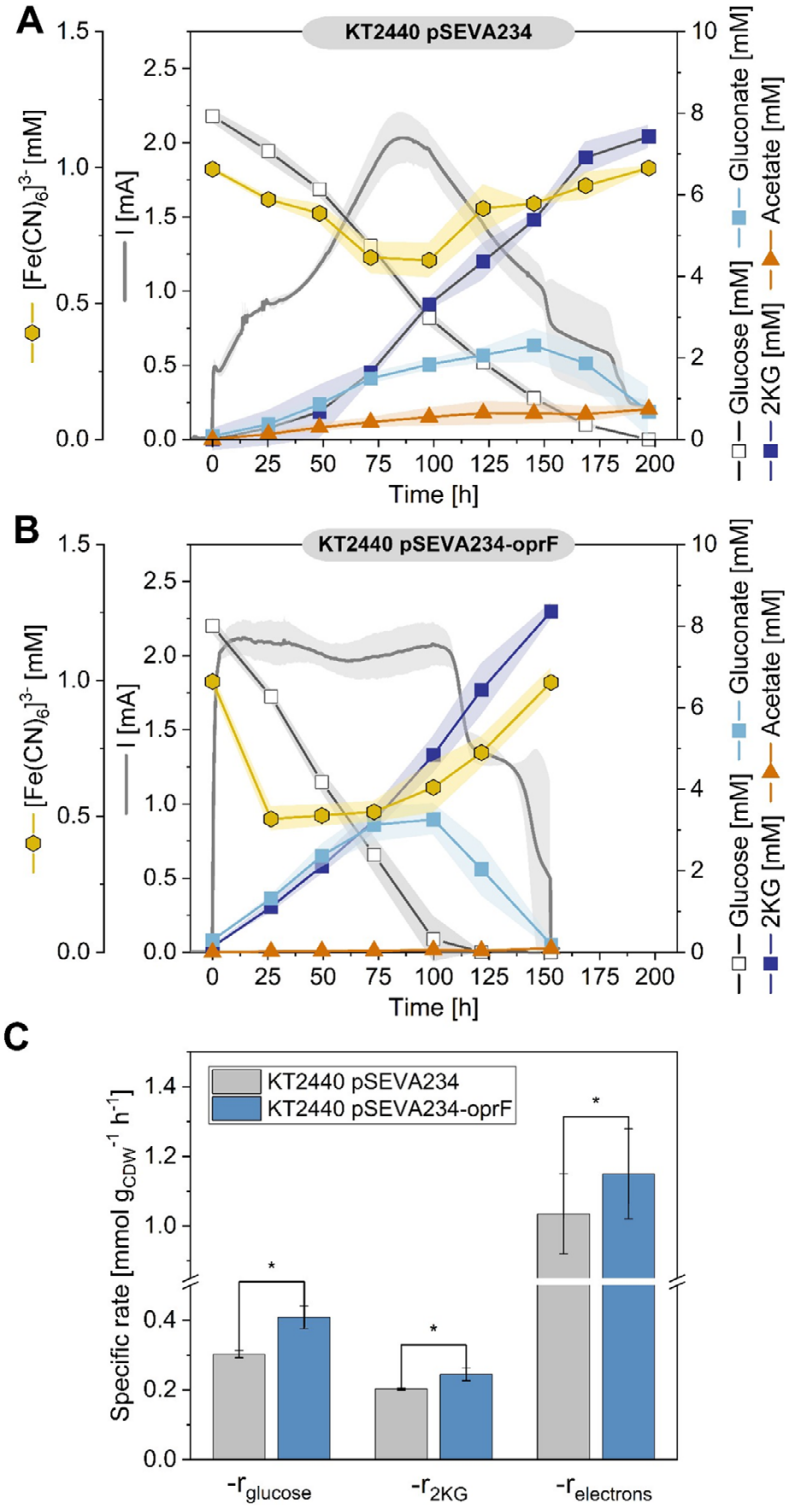
Impact of OprF overexpression on the bioelectrochemical performance of *P. putida* when [Fe(CN)_6_]^3−^ is used as the mediator. (A) Time‐course data showing current production (mA) and concentrations (mM) of glucose, gluconate, and 2‐ketogluconate (2KG) for *P. putida* KT2440 pSEVA234. (B) Corresponding data for *P. putida* KT2440 pSEVA234‐oprF. (C) Comparison of specific rates for glucose consumption, 2KG formation and electron transfer between *P. putida* KT2440 pSEVA234 (grey) and *P. putida* KT2440 pSEVA234‐oprF (blue). Statistical significance was assessed using Student's *t* test (*p* < 0.05, *), (*n* = 3).

OprF overexpression resulted in a 3.6‐fold increase in fluorescence intensity in the wild‐type *P. putida* strain (Figure 6A) and a 1.8‐fold increase in the *ΔexbBD ΔtonB* mutant (Figure 7B), indicating increased membrane permeability in both strains. With increased permeability, wild‐type *P. putida* exhibited a notable increase in the mediator reduction rate in anaerobic serum flasks containing 1 mM ferricyanide, increasing from 0.05 ± 0.01 mM/h to 0.17 ± 0.01 mM/h. In contrast, *P. putida ΔexbBD ΔtonB* showed no increase in the reduction rate despite its increased permeability (Figure 6C,D). If passive diffusion through porins was one of the primary mediator uptake mechanisms, then strengthening passive diffusion by increasing external mediator concentrations (Figure 4B) or porin abundance (Figure 6) would be expected to increase periplasmic mediator levels and, consequently, EET rates, both in the presence and absence of the TonB system. However, our results using the *ΔexbBD ΔtonB* mutant support the conclusion that TonB‐dependent transport is required to achieve increased mediator uptake and EET rates in *P. putida* . These results suggest that while porin‐mediated diffusion likely contributes to basal mediator entry and efflux, TonB‐dependent transport is the main mechanism for mediator uptake.

### 2.6 Enhanced Anaerobic Performance Through the Overexpression of the Porin OprF in a BES

The overexpression of OprF led to a more than threefold increase in the mediator reduction rate in *P. putida* cultivated in serum flasks (Figure 6C). To further evaluate its impact, the OprF‐overexpressing strain was tested in a bioelectrochemical system and compared to *P. putida* carrying the empty plasmid pSEVA234. In the bioelectrochemical system, the control strain ( *P. putida* pSEVA234) required approximately 200 h to complete fermentation. The cells reached a peak current of 2.0 ± 0.17 mA after 85 h, with 2KG as the main product (Figure 7A). Interestingly, at the beginning of the process (0 h), the OprF‐overexpressing strain presented a greater initial current (2.1 mA vs. 0.5 mA in the control), which indicates improved initial mediator accessibility and accelerated extracellular electron transfer between the cells and the anode (Figure 7B). Consistently, the specific electron transfer rate significantly increased from 1.035 ± 0.115 mmol g_CDW_^−1^ h^−1^ in the control strain to 1.15 ± 0.129 mmol g_CDW_^−1^ h^−1^ in the OprF‐overexpressing strain (Figure 7C). The current remained elevated until the glucose was fully consumed. Notably, a second current phase emerged between 125 and 150 h, during which the accumulated gluconate was completely converted to 2KG, although with a lower current. Overall, the OprF‐overexpressing strain presented greater glucose consumption, a reduction in byproduct formation (acetate), and higher 2KG production rates than did *P. putida* pSEVA234, making it a valuable cell factory for bioelectrochemical application (Figure 7).

## 3 Discussion

### 3.1 Selective Bacterial Membrane Permeability and Its Impact on Mediated Extracellular Electron Transfer

The outer membrane of gram‐negative bacteria serves as a selective barrier, limiting the unrestricted entry of harmful substances while allowing for the passage of essential nutrients. This selective permeability presents a challenge for mediator‐based EET in BESs since the molecular interaction sites of employed mediators for electron transfer are often located at the inner membrane (Gemünde et al. 2022) (Figure 2). For natural hydrophilic mediators such as riboflavin, proteins such as Bfe in *Shewanella oneidensis* and YeeO in *E. coli* (McAnulty and Wood 2014) are known to play roles in secretion, whereas several putative receptors and transporters are proposed to mediate riboflavin uptake (García‐Angulo 2017). However, the use of natural mediators presents challenges, including metabolic burden and stability issues (Chukwubuikem et al. 2021). In contrast, the transport mechanisms for exogenous hydrophilic mediators remain largely unknown. Identifying and targeting these transport systems is important for rationally improving mediated extracellular electron transfer.

Transport across the outer membrane can be divided into passive and active transport mechanisms. Passive transport (diffusion) relies on porins and substrate‐specific channels, where ß‐barrel membrane proteins enable molecule passage along the concentration gradient without requiring energy. OprF is the primary nonspecific porin in pseudomonads, with a higher exclusion limit (~3 kDa) than the major porins of *E. coli* (~600 Da) (Hancock and Nikaido 1978; Sugawara et al. 2006). In addition to enabling the diffusion of ions (weakly cation selective), sugars, nitrate/nitrite and toluene, OprF also contributes to maintaining cell shape (Bellido et al. 1992; Yoon et al. 2002; Chevalier et al. 2017; Poblete‐Castro et al. 2020). In contrast, active transport is essential for the uptake of compounds that are either too large to diffuse through porins or so scarce that diffusion rates are too low, enhancing survival and adaptability in dynamic environments by maintaining a stable supply of essential nutrients. This process occurs through TonB‐dependent receptors, which consist of a 22‐stranded β‐barrel with a pore that is blocked by an N‐terminal plug domain. For substrate uptake, the mechanically weak subdomain of the plug must unfold, a process driven by energy from the proton motive force at the inner membrane, which is transmitted via the TonB complex (Silale and van den Berg 2023).

### 3.2 Uptake of Hydrophilic Redox Mediators by the TonB System

In this study, we demonstrated that TonB‐dependent transport is important for the uptake of hydrophilic redox mediators across the outer membrane in *P. putida* KT2440 (Figure 1) and that deletion of the TonB complex resulted in impaired mediator reduction, a decreased EET rate, and reduced bioelectrochemical system performance (Figures 3, 4 and 5). While TonB‐dependent transport has been well studied in the context of iron uptake (Noinaj et al. 2010; Molina et al. 2016; Klebba et al. 2021; Silale and van den Berg 2023), its participation in inorganic metal mediator transport has not been explored. Interestingly, *P. putida* possesses numerous TonB‐dependent receptors that facilitate the transport of self‐secreted siderophores (iron‐chelating molecules, pyoverdine in the case of *P. putida* KT2440) but also exogenous heterologous siderophores from other microorganisms (xenosiderophores), such as enterobactin and desferroxamine from bacteria, ferrichrome from fungi, and ferric citrate from plant roots (Cornelis 2010). This broad uptake capability reflects the bacterium's ubiquity, adaptability, and strong potential for niche colonisation. The genome of *P. putida* harbours many nucleic acid sequences that encode extracytoplasmic function (ECF) sigma factors, and a large portion of these factors are dedicated to iron sensing and acquisition. Notably, 10 TBDRs in *P. putida* have been identified as similar to FecIR‐like clusters (Martínez‐Bueno et al. 2002). In *E. coli* , FecI is a sigma factor that regulates ferric citrate uptake, whereas FecR serves as its sensor (Enz et al. 2000). Our transcriptome data revealed significant upregulation of TBDRs (Figure 1), along with upregulation of several TBDR‐related ECF sigma factors (PP_3086: Log_2_‐FC 4.08, PP_4611: Log_2_‐FC 3.64, PP_4608: Log_2_‐FC 3.12, PP_3577: Log_2_‐FC 3.03, PP_0162: Log_2_‐FC 2.26, PP_0865: Log_2_‐FC 2.70, PP_1008: Log_2_‐FC 1.85, PP_0352: Log_2_‐FC 1.67, PP_0667: Log_2_‐FC 1.25, PP_2192: Log_2_‐FC 0.93). Given the broad substrate versatility of TBDRs and the significant upregulation of both TBDRs and TBDR‐related ECF sigma factors, artificial inorganic metal mediators such as [Fe(CN)_6_]^3−/4^ may be recognised and transported as metal–nutrient complexes.

### 3.3 Porins Might Contribute to Mediator Efflux

A lower yet detectable mediator reduction rate and current signal in the *ΔexbBD ΔtonB* mutant further suggest the presence of alternative mediator transport mechanism(s) (Figures 4 and 5). In addition to the participation of active TonB‐dependent transport, we demonstrated that increasing passive diffusion via the overexpression of the general porin OprF increased cell permeability and mediator reduction in the wild‐type strain, but it alone could not compensate for the loss of the TonB complex (Figures 6 and 7). Since no increase in reduced ferrocyanide levels was observed in the *ΔexbBD ΔtonB* mutant (Figure 6D), we propose that OprF primarily contributes to the efflux of ferrocyanide from the periplasm to the extracellular space. This would explain why its overexpression only increased ferricyanide reduction in the wild‐type strain, where TonB‐mediated uptake remains functional as the main mechanism for periplasmic mediator uptake. Notably, increased porin abundance could significantly reduce the time required for adaptation to the bioelectrochemical system in the presence of a functional TonB‐dependent transport system by increasing mediator flux, allowing for the system to operate immediately at full capacity (Figure 7). This effect may be further supported by increased glucose flux, similar to that observed in a glucose dehydrogenase (*gcd*)‐overexpressing *P. putida* strain (Yu et al. 2018), although the primary porin for glucose transport in *Pseudomonas* is OprB (Saravolac et al. 1991; Wylie and Worobec 1995; del Castillo et al. 2007). These findings support the idea that porins facilitate the nonspecific transport of various small polar compounds, including mediators and glucose.

An alternative strategy to enhance transmembrane mediator transport involves artificial membrane permeabilisation via agents such as branched polyethyleneimine (Soh et al. 2020) and cetyltrimethylammonium bromide (CTAB) (Wu et al. 2022; Gemünde et al. 2024). While effective in increasing cell permeability, these agents pose challenges, including potential membrane disruption and stress response induction, which may compromise cell viability over time. Consequently, more precise engineering strategies, such as those explored in this study, are preferable.

Future approaches should focus on optimising both passive and active transport mechanisms to improve the mediated electron transfer efficiency. Given that active transport via the TonB complex consumes proton motive force, efforts should prioritise the overexpression of specific TonB‐dependent receptors rather than the core energy complex TonB‐ExbD‐ExbB. This targeted strategy may lead to more efficient mediator transport while minimising energy costs.

## 4 Conclusion

The efficiency of mediated electron transfer relies on the continuous cycling of mediators between microbes and electrodes. Although the periplasmic redox protein essential for mediator reduction in *P. putida* has been identified, the mechanism by which the mediator is transported into the periplasm to access this native redox protein remains largely unknown. In this study, we identified the TonB complex as a key facilitator of transmembrane transport for inorganic metal complex mediators in *P. putida* . Additionally, we found that porin‐mediated diffusion serves as a secondary, passive transport mechanism. Notably, the presence of TonB was found to be essential for enhancing EET via OprF overexpression, further supporting the idea that TonB serves as the central mechanism for periplasmic mediator uptake. The OprF mutant immediately generated the maximum current output in the BES, whereas the control strain required 4 days to reach this level. In the future, comprehensive approaches such as omics data from the *ΔexbBD ΔtonB* mutant should be explored to identify the specific TonB‐dependent receptor(s), porin(s), and potentially active efflux systems involved in mediator cycling, among the many possible candidates. To further improve the anaerobic bioelectrochemical performance of *P. putida* , strain engineering efforts should focus on optimising the balance between passive porin‐mediated diffusion and active TonB‐dependent transport while fine‐tuning key electron‐producing pathways. In particular, enhancing the conversion of glucose to gluconate and 2‐ketogluconate (2KG) via glucose dehydrogenase (*Gcd*), which uses pyrroloquinoline quinone (PQQ) as a cofactor, and gluconate dehydrogenase (*Gad*), which further oxidises gluconate to 2KG, could improve efficiency (Kohlstedt et al. 2022). Previous studies have demonstrated that *gcd* overexpression and byproduct elimination enhance bioelectrochemical performance (Yu et al. 2018; Weimer et al. 2024). A deeper understanding of mediator transport not only enhances process efficiency but also minimises the reliance on high mediator concentrations, which can pose environmental risks (Lai et al. 2020) and complicate downstream removal (Fruehauf et al. 2020). By optimising mediator transport and metabolic function, *P. putida* can be further developed as a robust platform for sustainable bioelectrochemical applications.

## 5 Experimental Procedures

### 5.1 Bacterial Strains

*Pseudomonas putida* KT2440 was used as the wild‐type strain (Kohlstedt et al. 2018). *Escherichia coli* DH5α λpir (Biomedal Life Science, Seville, Spain), *E. coli* CC118 λpir (de Lorenzo and Timmis 1994), and *E. coli* HB101 (Sambrook et al. 1989) were used for cloning. A complete list of all the bacterial strains and plasmids used in this study is provided in Table 1. The oligonucleotide sequences used are listed in Table S2.

### 5.2 Genetic Engineering

Mutant derivatives of *P. putida* KT2440 were generated through chromosomal integration of the suicide plasmid pGNW2, followed by recombination mediated by the homing endonuclease I‐SceI encoded on pSEVA6231S (Wirth et al. 2020). The integrative plasmid, constructed via Gibson assembly, contained 500‐bp homologous regions flanking the target gene deletions. The plasmids were introduced into *P. putida* via tri‐parental mating. Extended passaging was necessary to eliminate the counterselection plasmid pSEVA6231S from the *P. putida ΔexbBD ΔtonB* strain, which resulted in a visible phenotypic change on LB plates characterised by a larger colony size. To exclude unintended genomic alterations, *P. putida ΔexbBD ΔtonB* carrying pSEVA6231S was used, with the wild‐type strain also transformed with pSEVA6231S for comparison. Deletions were confirmed by PCR and sequencing (GENEWIZ Azenta Life Sciences, Leipzig, Germany). The plasmid pSEVA234‐oprF was constructed using BamHI‐XbaI restriction sites and ligated with a Rapid DNA Ligation Kit (Thermo Fisher Scientific, Massachusetts, United States). The accuracy of the construct was verified by PCR and sequencing.

### 5.3 Media and Aerobic Culture Conditions

The cells were cultivated in defined M9 glucose medium (DM9) containing 5 g/L glucose (Lai et al. 2016). A single colony from a fresh LB agar plate was inoculated into a baffled shaker flask, ensuring a volume of less than 15% of the total flask volume. The cultures were incubated overnight (16 h) at 30°C and 230 rpm on a rotary shaker (Infors, Bottmingen, Switzerland). To increase the growth of *P. putida ΔexbBD ΔtonB*, the medium was supplemented with 20 μM FeCl_3_. If needed, precultures were supplemented with antibiotics and inducers at the following concentrations: gentamycin (30 μg mL^−1^), kanamycin (50 μg mL^−1^) and IPTG (1 mM). The main anaerobic cultures were grown without antibiotics or inducers, as the cells did not grow under these conditions.

### 5.4 Growth Rate Determination at Different Iron Concentrations

To assess the impact of iron availability on growth, wild‐type *P. putida* and the iron uptake‐deficient mutant *P. putida ΔexbBD ΔtonB* were cultured in DM9 supplemented with FeCl_3_ at concentrations of 10, 20 and 40 μM. Growth experiments were conducted in a bioreactor system with 48‐well FlowerPlates (Beckman Coulter GmbH, Baesweiler, Germany), with each well containing 1 mL of medium (Becker et al. 2018). The system was operated at 30°C, 1300 rpm, and 85% humidity. Cell growth was continuously monitored at OD_620_, and the maximum specific growth rate was determined by linear regression of ln(OD_620_) over time during the exponential growth phase. Three biological replicates were performed for each condition.

### 5.5 Serum Flask Experiments

The ferricyanide reduction rate was assessed in 30 mL serum bottles containing 25 mL of DM9 supplemented with 1.5 g/L glucose. The bottles were tightly sealed with aluminium caps in an N_2_ atmosphere to create anaerobic conditions (Lange et al. 2017). The cultures were incubated at 30°C and 180 rpm on a rotary shaker (Infors, Bottmingen, Switzerland). Three biological replicates were performed for each condition, and cell‐free bottles were used as controls.

### 5.6 Cell Culture Plate Experiments

To screen the mediator reduction rate under various conditions, experiments were conducted in 24‐well plates with different concentrations of [Fe(CN)_6_]^3−^ (0.5, 1.0, 2.0 and 4.0 mM) and FeCl_3_ (5.5 μM (basal level in DM9 medium), 15.5 and 25.5 μM). All conditions were prepared in DM9 medium supplemented with 1.5 g/L glucose and adjusted to a starting OD of 1. The prepared cultures were distributed across four separate well plates, with each condition set up in duplicate. An oxygen‐depleted atmosphere was established via an Anaerocult C mini (Merck, Millipore, Darmstadt, Germany). The plates were incubated at 30°C and 160 rpm on a rotary shaker (Infors, Bottmingen, Switzerland). To track mediator reduction over time, each plate was harvested at different time points, allowing for the generation of a time‐course dataset.

### 5.7 Bioelectrochemical System Setup and Electrochemical Analysis

The setup and operation of the bioelectrochemical system followed the methods described by (Lai et al. 2019). Briefly, the reactors were filled with 300 mL of DM9 (1.5 g/L glucose) supplemented with 1 mM of either K_3_[Fe(CN)_6_] or [Co(bpy)_3_](ClO_4_)_2_ as the mediator. The anode potential was set to 0.5 V versus AgCl/KCl (sat) when [Fe(CN)_6_]^3−/4‐^ was used and 0.3 V versus AgCl/KCl (sat) when [Co(bipy)_3_]^3+/2+^ was used. Strains harbouring pSEVA234 or pSEVA234‐oprF were first grown overnight (approximately 16 h) in DM9 medium (5 g/L glucose) and inoculated from a single colony on a fresh plate. The following day, the overnight culture was diluted to an initial OD_600_ of 0.2 to start a preculture. The preculture was grown until it reached an OD_600_ of 0.5–0.6 before it was induced with 1 mM IPTG and incubated for another 16 h before being used for the main culture, which had an initial OD_600_ of 0.8.

### 5.8 Quantification of Cells, Substrates, and Products

The cell concentration and oxidised mediator ([Fe(CN)_6_]^3−^) levels were measured spectrophotometrically at 600 nm (OD_600_) and 420 nm, respectively. The glucose concentration (Weiland et al. 2023) and the concentration of organic acids (Poblete‐Castro et al. 2013) were determined using high‐performance liquid chromatography (HPLC).

### 5.9 NPN Uptake Assay

Strains harbouring pSEVA234 or pSEVA234‐oprF were cultivated in minimal DM9 (5 g/L glucose) until they reached the middle of the exponential growth phase. Induction was initiated at an OD_600_ of 0.2 via the addition of 1 mM IPTG. The cells were harvested (1 mL, 5500 × *g*, 3 min), washed with PBS (pH 7.3), and centrifuged again. The resulting pellet was resuspended to a final OD_600_ of 0.4. For fluorescence intensity measurements, 200 μL of the cell suspension was mixed with 4 μL of NPN working solution (10 μM final concentration) in a black 96‐well plate. The NPN stock solution (5 mM) was prepared in acetone and diluted in PBS. The fluorescence intensity was measured immediately using a BioTek Synergy H1 Multimode Reader (BioTek, Vermont, United States), with an excitation wavelength of 350 nm and an emission range of 390–490 nm.

### 5.10 Expression Analysis of Outer Membrane Genes

Changes in the expression of outer membrane genes were analysed using previously published datasets (Weimer et al. 2024). The complete datasets are available in the GEO database (GSE266590) and the MassIVE repository (MSV000094887).

### 5.11 Data Processing and Statistical Analysis

The data presented in the figures are presented as the means ± standard deviations (SDs). Statistical analysis was conducted using Student's *t* test, with significance thresholds set at *p* < 0.05 (*) and *p* < 0.01 (**). All the statistical analyses were performed using OriginLab software (OriginPro 2023b, OriginLab Corporation, Northampton, MA, USA).

## Author Contributions

**Anna Weimer:** conceptualization, investigation, methodology, validation, visualization, writing – original draft, writing – review and editing, formal analysis. **Jens Krömer:** funding acquisition, writing – review and editing, resources, project administration, conceptualization. **Bin Lai:** conceptualization, funding acquisition, writing – review and editing, resources, project administration. **Christoph Wittmann:** conceptualization, funding acquisition, writing – original draft, writing – review and editing, visualization, validation, project administration, supervision, resources.

## Conflicts of Interest

The authors declare no conflicts of interest.

## Supporting information


**Table S1:** Expression of genes encoding proteins localised on the outer membrane (*p*_corr_ value < 0.05). Log FC > 2 are marked in yellow, Log FC < ‐2 are marked in blue. TonB complex (*tonB*, *exbBD*, TonB dependent receptors) are marked with, RND efflux protein genes are marked with, porins and porin‐like protein genes are marked with, and hypothetical protein genes are marked with.
**Table S2:** Primers used for constructing *P. putida* derivatives. Overhangs for Gibson assembly (pGNW2‐*∆*e*xbBD ∆tonB*, pGNW2‐*∆PP_1446*, pGNW2‐*∆PP_3325*) and enyzme restrictions (pSEVA234‐oprF) are underlined. RBS (Poblete‐Castro et al. 2020) in bold.
**Figure S1:** Reduction rate of [Fe(CN)_6_]^3−^. Reduction of [Fe(CN)_6_]^3−^ in anaerobic serum flask cultures (A) and the respective reduction rates (B) of the wild type (grey), and mutants of TBDR found significantly higher abundant in the transcriptome and proteome data set—ΔPP_1446 (yellow), ΔPP_3325 (orange), and ΔPP_1446 ΔPP_3325 (brown).
**Figure S2:** Impact of different mediator ([Fe(CN)_6_]^3−^) concentrations on the bio‐electrochemical performance of *P. putida* ∆*exbBD* ∆*tonB*. Time‐course data showing current density (mA/cm^2^) for *P. putida* ∆*exbBD* ∆*tonB* using different mediator concentration (0.5, 1, 2, 4, 10 mM).

## Data Availability

The data that support the findings of this study are available from the corresponding author upon reasonable request.
