# Supplementary material for: The TonB‐Dependent Transport System Facilitates the Uptake of Inorganic Metal Mediators in Pseudomonas putida KT2440 in a Bioelectrochemical System
Source: Microb Biotechnol. 2025 Aug 3;18(8):e70206. doi: 10.1111/1751-7915.70206 (PMC12319065; doi:10.1111/1751-7915.70206)
Supplement: Supplementary file 1 — Table S1: Expression of genes encoding proteins localised on the outer membrane (p corr value < 0.05). Log FC > 2 are marked in yellow, Log FC < ‐2 are marked in blue. TonB complex (tonB, exbBD, TonB dependent receptors) are marked with, RND efflux protein genes are marked with, porins and porin‐like protein genes are marked with, and hypothetical protein genes are marked with. Table S2: Primers used for constructing P. putida derivatives. Overhangs for Gibson assembly (pGNW2‐∆exbBD ∆tonB, pGNW2‐∆PP_1446, pGNW2‐∆PP_3325) and enyzme restrictions (pSEVA234‐oprF) are underlined. RBS (Poblete‐Castro et al. 2020) in bold. Figure S1: Reduction rate of [Fe(CN)6]3−. Reduction of [Fe(CN)6]3− in anaerobic serum flask cultures (A) and the respective reduction rates (B) of the wild type (grey), and mutants of TBDR found significantly higher abundant in the transcriptome and proteome data set—ΔPP_1446 (yellow), ΔPP_3325 (orange), and ΔPP_1446 ΔPP_3325 (brown). Figure S2: Impact of different mediator ([Fe(CN)6]3−) concentrations on the bio‐electrochemical performance of P. putida ∆exbBD ∆tonB. Time‐course data showing current density (mA/cm2) for P. putida ∆exbBD ∆tonB using different mediator concentration (0.5, 1, 2, 4, 10 mM). [file MBT2-18-e70206-s001.docx]

**1 Supporting Information**

**1.1 Tables**

**Table S. 1** **Expression of genes encoding proteins localized on the outer membrane (p_corr_ value <0.05)**. Log FC>2 are marked in yellow, Log FC<-2 are marked in blue. TonB complex (*tonB*, *exbBD*, TonB dependent receptors) are marked with , RND efflux protein genes are marked with , porins and porin-like protein genes are marked with , and hypothetical protein genes are marked with .

|  | **Locus Tag** | **Gene name** | **p_corr_ value** | **Log FC** | **Product Name (Uniprot)** |
| --- | --- | --- | --- | --- | --- |
|  | PP_2408 | *czcC-II* | 4.4E-07 | 4.80 | cobalt-zinc-cadmium resistance protein |
|  | PP_1046 | *xcpQ* | 3.1E-06 | 4.78 | type II secretion pathway protein XcpQ |
|  | PP_4755 |  | 7.5E-07 | 4.72 | ferrichrome-iron receptor |
|  | PP_1284 | *algE* | 4.4E-07 | 4.69 | alginate production protein AlgE |
|  | PP_3069 |  | 4.1E-07 | 4.63 | outer membrane autotransporter |
|  | PP_1044 | *uxpA* | 4.8E-07 | 4.62 | lipoprotein UxpA |
|  | PP_5385 | *czcC* | 8.1E-07 | 4.62 | CzcC family metal RND transporter outer membrane protein |
|  | PP_0037 | *oprP* | 4.2E-07 | 4.60 | porin P |
|  | PP_2590 |  | 1.9E-06 | 4.58 | ferric siderophore receptor |
|  | PP_3390 |  | 8.9E-07 | 4.58 | porin |
|  | PP_1173 | *galP-I* | 6.5E-07 | 4.54 | porin-like protein |
|  | PP_2819 | *oprJ* | 3.9E-07 | 4.53 | outer membrane protein OprJ |
|  | PP_2420 |  | 1.8E-06 | 4.50 | ferric siderophore receptor |
|  | PP_4613 | *fecA* | 5.1E-06 | 4.49 | outer membrane ferric citrate porin |
|  | PP_2193 |  | 7.2E-07 | 4.49 | ferric siderophore receptor |
|  | PP_3340 |  | 6.1E-07 | 4.48 | TonB-dependent receptor |
|  | PP_0046 | *opdT-I* | 3.8E-06 | 4.34 | tyrosine-specific outer membrane porin D |
|  | PP_2517 | *galP* | 3.0E-06 | 4.33 | porin-like protein |
|  | PP_1889 | *fimD* | 4.8E-06 | 4.32 | type I pili subunit FimD |
|  | PP_2058 |  | 1.4E-05 | 4.31 | porin |
|  | PP_0669 |  | 1.0E-06 | 4.30 | ferric siderophore receptor |
|  | PP_2042 |  | 3.6E-07 | 4.28 | hypothetical protein |
|  | PP_2702 |  | 8.0E-06 | 4.27 | porin |
|  | PP_2069 |  | 1.1E-05 | 4.26 | multidrug MFS transporter outer membrane protein |
|  | PP_0861 |  | 4.0E-06 | 4.25 | outer membrane ferric siderophore receptor |
|  | PP_1263 |  | 1.1E-05 | 4.24 | fusaric acid resistance protein |
|  | PP_4211 | *ompQ* | 2.0E-06 | 4.18 | outer membrane pyoverdine efflux protein |
|  | PP_3084 |  | 7.5E-06 | 4.16 | ferric siderophore receptor |
|  | PP_0350 |  | 9.7E-07 | 4.14 | ferrichrome-iron receptor |
|  | PP_3427 | *oprN* | 7.3E-07 | 4.04 | multidrug RND transporter outer membrane protein OprN |
|  | PP_3200 |  | 2.4E-05 | 4.03 | hypothetical protein |
|  | PP_2638 |  | 2.4E-06 | 4.00 | cellulose synthase operon protein C |
|  | PP_2204 | *copB-I* | 4.4E-06 | 3.99 | copper resistance protein B |
|  | PP_0267 |  | 1.7E-06 | 3.94 | ferric siderophore receptor |
|  | PP_3450 |  | 1.7E-06 | 3.93 | TPR repeat-containing protein |
|  | PP_0045 | *czcC-I* | 6.5E-07 | 3.92 | cobalt-zinc-cadmium resistance protein |
|  | PP_3373 | *bamA-II* | 1.6E-05 | 3.90 | outer membrane protein assembly factor |
|  | PP_3478 |  | 1.4E-06 | 3.90 | secretion protein |
|  | PP_2242 | *fepA* | 4.8E-06 | 3.89 | ferric enterobactin transport system outer membrane subunit |
|  | PP_1450 |  | 1.4E-06 | 3.75 | TPS family activation/secretion protein |
|  | PP_4971 |  | 2.0E-05 | 3.67 | outer membrane-bound lytic murein transglycolase A |
|  | PP_0867 |  | 2.4E-04 | 3.67 | FecA-like outer membrane receptor |
|  | PP_4544 |  | 4.7E-06 | 3.63 | hypothetical protein |
|  | PP_1880 |  | 2.1E-06 | 3.59 | outer membrane autotransporter |
|  | PP_3299 |  | 9.4E-07 | 3.57 | lipoprotein |
|  | PP_3612 |  | 8.4E-06 | 3.56 | TonB-dependent receptor |
|  | PP_3271 | *phaK* | 3.2E-06 | 3.52 | phenylacetic acid-specific porin |
|  | PP_3464 |  | 3.9E-05 | 3.45 | hypothetical protein |
|  | PP_0179 |  | 1.4E-06 | 3.41 | putative efflux transporter |
|  | PP_3764 | *opdN* | 1.4E-03 | 3.36 | outer membrane porin D |
|  | PP_4291 |  | 3.1E-05 | 3.29 | hypothetical protein |
|  | PP_1847 |  | 7.5E-06 | 3.28 | TonB-dependent ferric siderophore receptor |
|  | PP_3939 | *nicP-II* | 1.6E-06 | 3.25 | porin-like protein |
|  | PP_0160 |  | 2.6E-05 | 3.12 | ferrioxamine receptor |
|  | PP_1006 |  | 1.2E-05 | 3.11 | heme receptor |
|  | PP_0272 |  | 3.0E-05 | 3.04 | ferric siderophore receptor |
|  | PP_4217 | *fpvA* | 1.5E-02 | 2.97 | TonB-dependent outer membrane ferripyoverdine receptor FpvA |
|  | PP_2662 |  | 1.2E-05 | 2.97 | hypothetical protein |
|  | PP_3575 |  | 2.1E-04 | 2.95 | ferric siderophore receptor |
|  | PP_3330 |  | 1.4E-04 | 2.92 | ferric siderophore receptor |
|  | PP_3155 |  | 2.1E-05 | 2.92 | ferric siderophore receptor |
|  | PP_0535 |  | 2.0E-05 | 2.84 | ferric siderophore receptor |
|  | PP_3570 | *oprB-III* | 3.0E-05 | 2.73 | carbohydrate-selective porin |
|  | PP_3325 |  | 1.7E-03 | 2.67 | ferric siderophore receptor |
|  | PP_4137 |  | 8.5E-06 | 2.67 | outer membrane siderophore receptor |
|  | PP_5250 | *opdB* | 1.9E-05 | 2.60 | proline-specific outer membrane porin D |
|  | PP_3656 |  | 1.4E-05 | 2.56 | aromatic compound-specific porin |
|  | PP_1446 |  | 2.0E-02 | 2.44 | TonB-dependent receptor |
|  | PP_3630 | *opdT-II* | 2.6E-04 | 2.38 | tyrosine-specific outer membrane porin D |
|  | PP_0573 |  | 6.3E-05 | 2.38 | hypothetical protein |
|  | PP_4606 |  | 9.0E-05 | 2.31 | ferric siderophore receptor |
|  | PP_1449 |  | 5.5E-04 | 2.29 | hypothetical protein |
|  | PP_3582 |  | 1.9E-05 | 2.25 | RND transporter outer membrane protein |
|  | PP_4145 | *mltD* | 2.5E-05 | 2.14 | membrane-bound lytic murein transglycosylase D |
|  | PP_4514 |  | 1.7E-04 | 2.09 | alpha/beta hydrolase superfamily esterase |
|  | PP_3397 |  | 1.7E-05 | 1.97 | hypothetical protein |
|  | PP_1061 |  | 8.4E-05 | 1.95 | ATP-dependent DNA helicase |
|  | PP_1445 | *oprB-II* | 2.3E-04 | 1.90 | carbohydrate-selective porin |
|  | PP_1577 |  | 1.3E-04 | 1.90 | lambda family tail tape meausure protein |
|  | PP_0418 | *estP* | 1.7E-03 | 1.88 | esterase EstP |
|  | PP_4032 |  | 3.3E-04 | 1.87 | lipoprotein Blc |
|  | PP_1502 |  | 1.1E-03 | 1.75 | OmpA family protein |
|  | PP_4057 |  | 1.0E-02 | 1.74 | membrane protein |
|  | PP_3168 | *nicP-I* | 4.0E-03 | 1.73 | porin-like protein |
|  | PP_1384 | *ttgC* | 1.6E-03 | 1.70 | efflux pump outer membrane protein TtgC |
|  | PP_0504 | *oprG* | 1.1E-05 | 1.68 | outer membrane protein OprG |
|  | PP_4989 | *pilJ* | 5.8E-05 | 1.68 | twitching motility protein PilJ |
|  | PP_1798 |  | 7.0E-04 | 1.66 | outer membrane efflux protein |
|  | PP_0799 | *opdC* | 1.5E-05 | 1.61 | histidine-specific outer membrane porin D |
|  | PP_1599 | *bamA-I* | 1.6E-04 | 1.58 | outer membrane protein assembly factor |
|  | PP_2754 |  | 1.7E-04 | 1.54 | OprD family outer membrane porin |
|  | PP_2892 |  | 1.0E-04 | 1.50 | hypothetical protein |
|  | PP_1579 |  | 5.5E-03 | 1.40 | hypothetical protein |
|  | PP_1887 |  | 1.6E-02 | 1.37 | hypothetical protein |
|  | PP_1273 |  | 1.0E-03 | 1.29 | multidrug MFS transporter outer membrane protein |
|  | PP_1419 | *opdH* | 7.2E-03 | 1.11 | tricarboxylate-specific outer membrane porin |
|  | PP_0913 |  | 3.5E-02 | 1.07 | hypothetical protein |
|  | PP_5308 | *tonB* | 1.1E-02 | 0.97 | TonB energy transducing system subunit TonB |
|  | PP_5057 |  | 2.8E-04 | 0.93 | M23/M37 family peptidase |
|  | PP_0678 |  | 1.9E-02 | 0.93 | hypothetical protein |
|  | PP_0525 |  | 1.9E-03 | 0.84 | B12 family TonB-dependent receptor |
|  | PP_1383 | *galP-II* | 4.5E-04 | 0.81 | porin-like protein |
|  | PP_2558 |  | 2.3E-02 | 0.73 | outer membrane efflux protein |
|  | PP_4171 |  | 3.6E-03 | 0.72 | hypothetical protein |
|  | PP_0715 |  | 1.5E-02 | 0.65 | outer membrane efflux protein |
|  | PP_0773 | *yiaD* | 3.0E-02 | 0.49 | OmpA/MotB domain-containing protein |
|  | PP_5037 |  | 3.6E-02 | 0.48 | lipocalin family lipoprotein |
|  | PP_0938 |  | 1.1E-04 | 0.43 | hypothetical protein |
|  | PP_3852 |  | 2.7E-03 | 0.26 | BNR domain-containing protein |
|  | PP_0805 |  | 2.8E-02 | 0.19 | outer membrane efflux protein |
|  | PP_4669 |  | 2.0E-02 | -0.19 | OmpA family protein |
|  | PP_5379 | *copB-II* | 3.1E-02 | -0.37 | copper resistance protein B |
|  | PP_5307 | *exbD* | 1.1E-02 | -0.46 | TonB-gated outer membrane transporter gating inner membrane protein |
|  | PP_5306 | *exbB* | 5.4E-03 | -0.55 | biopolymer transport protein ExbB |
|  | PP_0577 |  | 9.6E-03 | -0.58 | hypothetical protein |
|  | PP_4303 |  | 4.5E-03 | -0.88 | hypothetical protein |
|  | PP_5080 | *pilQ* | 9.5E-04 | -0.99 | type IV pili biogenesis protein |
|  | PP_0192 | *fkl* | 5.1E-04 | -1.01 | FKBP-type peptidyl-prolyl cis-trans isomerase |
|  | PP_0851 | *pilF* | 2.3E-04 | -1.05 | type IV pili biogenesis protein PilF |
|  | PP_0765 |  | 1.7E-02 | -1.13 | hypothetical protein |
|  | PP_4293 |  | 8.2E-05 | -1.14 | hypothetical protein |
|  | PP_2089 | *oprF* | 4.3E-02 | -1.15 | porin F |
|  | PP_4465 |  | 7.0E-03 | -1.20 | porin |
|  | PP_4923 |  | 1.4E-02 | -1.24 | outer membrane efflux protein |
|  | PP_1689 |  | 2.5E-02 | -1.33 | long-chain fatty acid transporter |
|  | PP_1131 | *slyB* | 1.9E-04 | -1.35 | outer membrane lipoprotein |
|  | PP_1087 |  | 4.8E-03 | -1.39 | OmpA family outer membrane protein |
|  | PP_0329 |  | 4.2E-04 | -1.43 | hypothetical protein |
|  | PP_4704 |  | 6.8E-05 | -1.43 | hypothetical protein |
|  | PP_1122 |  | 8.8E-05 | -1.60 | OmpA family protein |
|  | PP_1019 | *oprB-I* | 4.9E-02 | -1.72 | carbohydrate-selective porin |
|  | PP_1622 |  | 1.4E-05 | -1.88 | M23B subfamily metallopeptidase |
|  | PP_0883 | *opdP* | 6.6E-03 | -1.94 | glycine-glutamate dipeptide porin |
|  | PP_4384 | *flgH* | 3.2E-05 | -2.02 | flagellar L-ring protein |
|  | PP_2163 | *vacJ* | 7.2E-06 | -2.22 | lipoprotein VacJ |
|  | PP_1206 | *oprD* | 8.9E-04 | -2.29 | basic amino acid specific porin OprD |
|  | PP_3214 |  | 3.8E-07 | -3.19 | hypothetical protein |

**Table S. 2 Primers used for constructing *P. putida* derivatives.** Overhangs for Gibson assembly (pGNW2-*∆*e*xbBD* *∆tonB*, pGNW2-*∆PP_1446*, pGNW2-*∆PP_3325*) and enyzme restrictions (pSEVA234-oprF) are underlined. RBS ([Poblete-Castro et al., 2020](#_ENREF_1)) in bold.

| **Primer name** | **Sequence** |
| --- | --- |
| ∆*exbBD* ∆*tonB* -TS1-fwd | GTCGACTCTAGAGGATCCCCCTGCTTGAGACGAACAGCAG |
| ∆*exbBD* ∆*tonB* - TS1-rev | TTGTGACAGAAGCAAAAGTCGCTGGCCGGACCTGATGAA |
| ∆*exbBD* ∆*tonB* -TS2-fwd | TTCATCAGGTCCGGCCAGCGACTTTTGCTTCTGTCACAA |
| ∆*exbBD* ∆*tonB* -TS2-rev | TGAATTCGAGCTCGGTACCCGTTGCGCAGCTTTTCACG |
| ∆*PP_1446* -TS1-fwd | GGTCGACTCTAGAGGATCCCCACCAATCAGGCTGGCCT |
| ∆*PP_1446* -TS1-rev | CGATCTTCGTCGCTTGTGGTGGGGATGGGGCGTTCCTTG |
| ∆*PP_1446* – TS2-fwd | CCAAGGAACGCCCCATCCCCACCACAAGCGACGAAGATCG |
| ∆*PP_1446* -TS2-rev | TGAATTCGAGCTCGGTACCCGTCAGCTGGAATTCGGTGTC |
| ∆*PP_3325* -TS1-fwd | ATGCATGATGGTTCCTCGGTGTAAAGTCTCCGTTTTTCACG |
| ∆*PP_3325* -TS1-rev | GAATTCGAGCTCGGTACCCTGGGCAGAACCTGATGTTC |
| ∆*PP_3325* – TS2-fwd | GTCGACTCTAGAGGATCCCCCCTGCACTTCCAGCGTCTTGC |
| ∆*PP_3325* -TS2-rev | GTGAAAAACGGAGACTTTACACCGAGGAACCATCATGCAT |
| pSEVA234-oprF*_*fwd | TAAGCA*GGATCC***AGGAGGAAAAACAT**ATGAAACTGAAAAACACCTTGG |
| pSEVA234-oprF*_*rev | TAAGCA*TCTAGA*TTACTTGGCCTGGGCTTCTA |

**1.2 Figures**


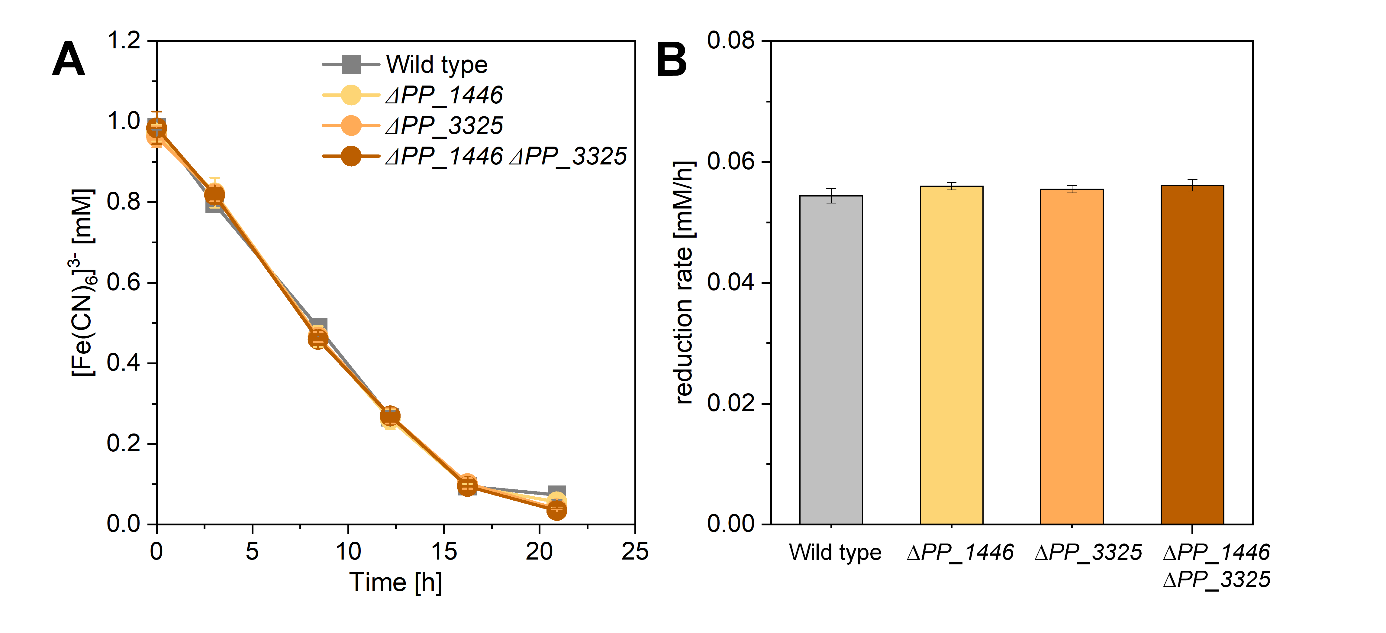


**Figure S. 1 Reduction rate of [Fe(CN)_6_]^3-^.** Reduction of [Fe(CN)_6_]^3-^ in anaerobic serum flask cultures (A) and the respective reduction rates (B) of the wild type (grey), and mutants of TBDR found significantly higher abundant in the transcriptome and proteome data set - ΔPP_1446 (yellow), ΔPP_3325 (orange), and ΔPP_1446 ΔPP_3325 (brown).

**
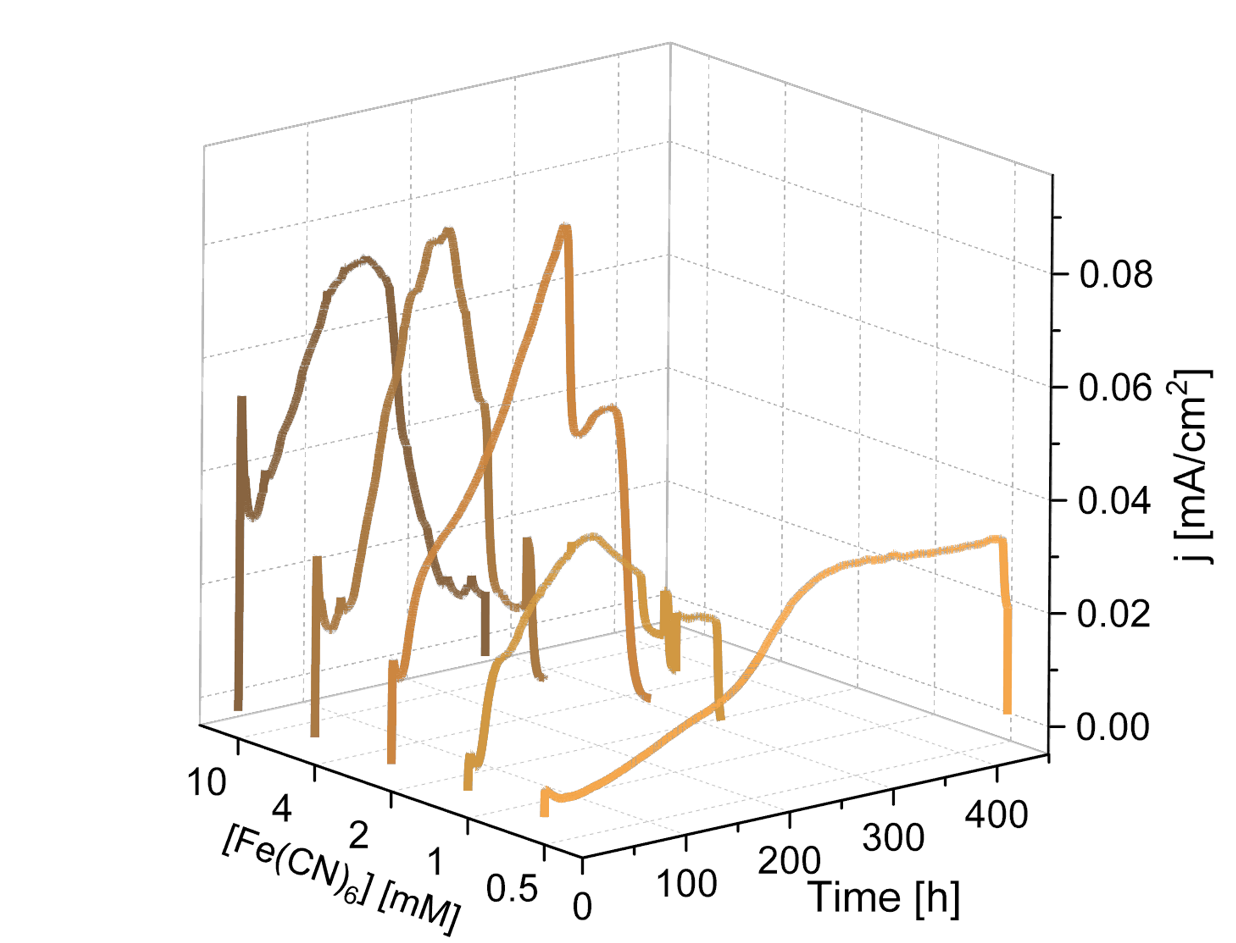
**

**Figure S2. Impact of different mediator ([Fe(CN)_6_]^3-^) concentrations on the bio-electrochemical performance of *P. putida* ∆*exbBD* ∆*tonB*.** Time-course data showing current density (mA/cm^2^) for *P. putida* ∆*exbBD* ∆*tonB* using different mediator concentration (0.5, 1, 2, 4, 10 mM).

**References**

Poblete-Castro, I., Aravena-Carrasco, C., Orellana-Saez, M., Pacheco, N., Cabrera, A., & Borrero-de Acuña, J. M. (2020). Engineering the Osmotic State of *Pseudomonas putida* KT2440 for Efficient Cell Disruption and Downstream Processing of Poly(3-Hydroxyalkanoates). *Frontiers in Bioengineering and Biotechnology, 8*, 161-161.
